# Primary care treatment guidelines for skin infections in Europe: congruence with antimicrobial resistance found in commensal *Staphylococcus aureus* in the community

**DOI:** 10.1186/s12875-014-0175-8

**Published:** 2014-10-25

**Authors:** Evelien ME van Bijnen, W John Paget, Casper DJ den Heijer, Ellen E Stobberingh, Cathrien A Bruggeman, François G Schellevis, in collaboration with the APRES Study Team

**Affiliations:** Netherlands Institute for Health Services Research (NIVEL), Otterstraat 118-124, Utrecht, The Netherlands; Department of Medical Microbiology/School for Public Health and Primary Care (CAPHRI), Maastricht University Medical Centre, P. Debyelaan 25, Maastricht, The Netherlands; Department of General Practice and Elderly Care Medicine/EMGO Institute for Health and Care Research, VU University Medical Centre, De Boelelaan 1117, Amsterdam, The Netherlands

**Keywords:** Antibiotic resistance, Treatment guidelines, Primary care, Skin infections

## Abstract

**Background:**

Over 90% of antibiotics for human use in Europe are prescribed in primary care. We assessed the congruence between primary care treatment guidelines for skin infections and commensal *Staphylococcus aureus* (*S. aureus*) antimicrobial resistance levels in community-dwelling persons.

**Methods:**

The prevalence of antimicrobial resistance in *S. aureus* was analysed by taking nose swabs from healthy primary care patients in nine European countries (total N = 32,032). Primary care treatment guidelines for bacterial skin infections were interpreted with respect to these antimicrobial resistance patterns. First- and second-choice recommendations were assessed and considered congruent if resistance to the antibiotic did not exceed 20%.

**Results:**

We included primary care treatment guidelines for impetigo, cellulitis, folliculitis and furuncle. Treatment recommendations in all countries were consistent: most of the first-choice recommendations were beta-lactams, both for children and adults. Antimicrobial resistance levels were low, except for penicillin (on average 73% resistance). Considerable variation in antimicrobial resistance levels was found between countries, with Sweden displaying the lowest levels and Spain the highest. In some countries resistance to penicillin and azithromycin was significantly higher in children (4-17 years) compared with adults.

**Conclusions:**

Most of the first- and second-choice recommendations in the treatment guidelines for skin infections were congruent with commensal *S. aureus* antimicrobial resistance patterns in the community, except for two recommendations for penicillin. Given the variation in antimicrobial resistance levels between countries, age groups and health care settings, national data regarding antimicrobial resistance in the community should be taken into account when updating or developing primary care treatment guidelines.

**Electronic supplementary material:**

The online version of this article (doi:10.1186/s12875-014-0175-8) contains supplementary material, which is available to authorized users.

## Background

Antimicrobial resistance (AMR) has become an important public health threat across the globe during recent decades [1–3]. The development of AMR is considered to be mainly driven by antibiotic use: exposure to antibiotics leads to the selection of resistant bacteria in the commensal microbiota [4–6]. An important source of exposure is found in primary care as over 90% of all antibiotics for human medical use in Europe are prescribed in primary care [5,7]. Therefore, several studies have advocated cautious and appropriate prescribing of antibiotics to control the emergence of AMR [1,8]: empirical treatment with antibiotics should only take place if necessary and should ideally include appropriate agents which are effective against the most common pathogenic bacteria [8].

An inappropriate antibiotic treatment will have several effects, in the first place for the patient: the effectiveness of the treatment will be limited. Secondly, unnecessary costs will occur for the health care system; and finally, the exposure to antibiotics could lead to a further increase of AMR [1,9,10]. Several studies recommend the use of relevant AMR data when developing or revising primary care treatment guidelines for bacterial infections [6,8,11]. However, since previous AMR studies have mainly obtained data from hospitalized populations with higher resistance levels [6], primary care treatment guidelines might benefit by integrating AMR patterns from the community [12,13].

*S. aureus* is a part of the commensal microbiota mainly manifesting as bacterial skin and soft tissue infections [14,15]. The incidence of these infections in primary care is relatively high, especially in children, hereby forming a considerable cause for antibiotic prescriptions [14,16,17]. Traditionally, methicillin-resistant *S. aureus* (MRSA) was confined to hospitals and long-term-care facilities, but in the last decade MRSA infections have also appeared in healthy community-dwelling individuals [18–21]. Several studies have established the importance of commensal microbiota as a natural reservoir of bacterial resistance, from which resistance can be acquired by pathogens [22,23]. By focusing on *S. aureus*, our study assessed the congruency of primary care treatment guidelines for skin infections with AMR data from the community, to optimize treatment effectiveness.

## Methods

### Study design

This study was part of the EC-funded APRES study, aimed at establishing the appropriateness of prescribing antibiotics in primary care in Europe, by collecting data on AMR in the community, antibiotic prescription behaviour and treatment guidelines in primary care. Nine countries across Europe participated in APRES, with varying patterns of antibiotic prescription rates [5]: Austria, Belgium, Croatia, France, Hungary, the Netherlands, Spain, Sweden, and the United Kingdom. A detailed overview of the APRES study design and an analysis of the AMR results have been published elsewhere [4,24]. This paper relates the measured AMR patterns in the community to primary care treatment guidelines for skin infections and assesses their congruency.

### Study participants and AMR

In each of the nine countries, national GP networks selected 20 primary care practices representative of their total GP population. From each of these practices 200 nasal swabs from patients visiting the practice for non-infectious reasons were collected [4,24]. Previous studies [25,26] have shown carriage of *S. aureus* to be dynamic and occurring on multiple bodily sites. With the nares being a common site for *S. aureus* we assumed our sample to be representative of all carriage. In order to assess AMR levels in the commensal flora in the community (from which resistance can be acquired by pathogens), we excluded patients with known important risk factors for AMR: antibiotic use or hospitalisation in the past 3 months. Although *S. aureus* is not the sole pathogen causing skin infections, we selected it due to its impact on public health and relatively high nasal carriage rate [25,26]. After isolation of *S. aureus* in 8 national laboratories using standardised procedures, we determined in one central laboratory whether the isolates were resistant or susceptible to a range of commonly used antibiotics in primary care, using cut-off points from the Eucast guidelines [4,27].

### Treatment guidelines for skin infections

Coordinators of national GP networks in each participating country supplied the most commonly used and most recent primary care treatment guidelines for bacterial skin infections. With the exception of Croatia, all countries had issued national treatment guidelines for one or more bacterial skin infections. This resulted in a total of 13 national guidelines from 8 European countries (see Additional file 1: Table S1), from which we extracted the prescription recommendations. We focused the analysis on the antibiotic prescription recommendations for four common bacterial skin infections in primary care which are often caused by *S. aureus* [13]: impetigo, cellulitis, folliculitis and furuncle. We have analysed the treatment recommendations for antibiotic therapy, distinguishing between first-choice recommendations and, if available, second-choice options. Since skin infections are common in children, we assessed the recommendations for children separately if this information was available.

### Data analysis

To assess the treatment guidelines issued on a national level, resistance levels for each antibiotic were aggregated to a national level by dividing the number of resistant *S. aureus* isolates per country by the total number of persons who carried a *S. aureus*. Separate rates were calculated for children (4-17 years old) and adults (18+), since treatment recommendations are often adapted for children. The recommended antibiotics in the treatment guideline were linked to the respective AMR levels in that country. Based on research regarding urinary tract infections, the antibiotic treatment recommendations were considered to be congruent if the resistance to the antibiotic did not exceed 20% [28]. Carriership of *S. aureus* is linked to a higher risk of bacterial skin infection [25,29], however, evidence on the relationship between nasal *S. aureus* and pathogenic *S. aureus* isolated from skin infections is lacking. Therefore, in our current comparison we assumed that pathogenic *S. aureus* related to skin infections shows the same AMR patterns as nasal colonized *S. aureus*.

Not all antibiotics mentioned in the treatment guidelines were covered on a product level in the resistance testing of our study. When no AMR data for a specific antibiotic was available, we used two expert opinions (a medical microbiologist and pharmacist) to identify a similar antibiotic: e.g. since no clarithromycin resistance was tested we used data of azithromycin resistance (see Additional file 2: Table S2). Recent studies have shown that resistance to similar antibiotics can serve as a reliable indicator for the level of resistance to the original antibiotic [30,31].

## Results

Data were obtained from a total number of 32,206 swabs and in twenty-two percent (N = 6,956) *S. aureus* was present. After excluding patients for whom age was unknown 6037 (87,8%) adults (aged 18+) and 840 (12,2%) children (aged 4 to 17) were included in our study sample, of which 56% were female.

### Prevalence of resistance

Table 1 (adults) and Table 2 (children) show the AMR levels of *S. aureus* for five antibiotics per country. The difference in resistance was high: on average, *S. aureus* showed almost no resistance to oxacillin (0.4%) while resistance to penicillin was high (73%). Resistance to topical antibiotics was low: averaging 0.4% for mupirocin and 2.8% for fusidic acid. The level of variation between countries was considerable, especially regarding AMR levels to azithromycin which ranged from 1.5% in Sweden to 16.9% in France. Sweden stood out with the lowest AMR levels to all but one antibiotic. For azithromycin and penicillin significant differences between adults and children were found in some countries, with the higher resistance levels found in children.

**Table 1:** **Resistance rates of** ***S. aureus*** **isolates in nine European countries** – **adults 18**+

			**Resistance rates** **(%)** **(95%** **confidence interval)**						
**Country**	**Swabs**	**Isolates of** ***S. aureus***	**Azithromycin**	**Clindamycin**	**Erythromycin**	**Oxacillin**	**Penicillin**	**Fucidic acid**	**Mupirocin**
**Austria**	3168	522 (16.5%)	12.8** (6.3-19.4)	11.1 (4.9 - 17.3)	12.6** (6.1-19.1)	1.5 (0-3.9)	64.4 (55-73.8)	1.0 (0-3.0)	0.2 (0-1.1)
**Belgium**	2892	552 (19.1%)	16.3 (9.1-23.5)	14.4 (7.5-21.3)	16.3 (9.1-23.5)	2.2 (0-5.1)	72.5 (63.7-81.3)	3.4 (0-7.0)	0.4 (0-1.6)
**Croatia**	3380	601 (17.8%)	5.8 (1.2-10.4)	5.5 (1-10)	5.8 (1.2-10.4)	2.3 (0-5.2)	75.4** (67-83.8)	0.2 (0-1.1)	0.7 (0-2.3)
**France**	3536	777 (22.0%)	17.5 (10.1-25.0)	14.9* (7.9-21.9)	17.1 (9.7-24.5)	1.8 (0-4.4)	74.4 (65.8-83)	4.0 (0.2-7.8)	0.1 (0-0.7)
**Hungary**	2883	359 (12.5%)	10.3** (4.3-16.3)	10.3** (4.3-16.3)	10.3** (4.3-16.3)	1.9 (0-4.6)	71.0** (62.1-79.9)	0.3 (0-1.4)	0.3 (0-1.4)
**NL**	3491	947 (27.1%)	6.9 (1.9-11.9)	5.2 (0.8-9.6)	5.5 (1.0-10.0)	1.0 (0-3.0)	68.4 (59.3-77.5)	5.2 (0.8-9.6)	0
**Spain**	3563	620 (17.5%)	11.5 (5.3-17.8)	9.5 (3.8-15.2)	11.0 (4.9-17.1)	1.3 (0-3.5)	86.0* (79.2-92.8)	1.1 (0-3.1)	1.6 (0-4.1)
**Sweden**	2859	846 (29.6%)	1.3 (0-3.5)	11.8 (5.5-18.1)	13.0 (6.4-19.6)	0	64.3* (54.9-73.7)	1.9 (0-4.6)	0
**UK**	3152	811 (25.7%)	8.6 (3.1-14.1)	7.5 (2.3-12.7)	8.9 (3.3-14.5)	1.6 (0-4.1)	73.4 (64.7-82.1)	7.8 (2.5-13.1)	0

**Significant difference with children under p < 0.05.

*Significant difference with children under p < 0.1.

**Table 2:** **Resistance rates of** ***S. aureus*** **isolates in nine European countries** – **children** <**18**

			**Resistance rates (%) (95% confidence intervals)**						
**Country**	**Swabs**	**Isolates of** ***S. aureus***	**Azithromycin**	**Clindamycin**	**Erythromycin**	**Oxacillin**	**Penicillin**	**Fucidic acid**	**Mupirocin**
**Austria**	111	23 (20.7%)	30.4** (21.4-39.4)	13.0 (6.4-19.6)	30.4** (21.4-39.4)	0	73.9 (65.3-82.5)	4.3 (0.3-8.3)	0
**Belgium**	101	30 (22.9%)	16.7 (9.4-24)	16.6 (9.3-23.9)	16.6 (9.3-23.9)	0	63.3 (53.9-72.7)	0	0
**Croatia**	562	152 (27.0%)	5.3 (0.9-9.7)	4.6 (0.5-8.7)	5.3 (0.9-9.7)	0.7 (0-2.3)	88.2** (81.9-94.5)	0	0
**France**	309	94 (30.4%)	11.7 (5.4-18.0)	8.5 (3.0-14.0)	10.6 (4.6-16.6)	1.1 (0-3.1)	79.8 (71.9-87.7)	2.1 (0-4.9)	0
**Hungary**	930	171 (18.4%)	16.4** (9.1-23.7)	16.4 (9.1-23.7)	16.4 (9.1-23.7)	0.6 (0-2.1)	86.0** (79.2-92.8)	0	0
**NL**	323	119 (36.8%)	5.9 (1.3-10.5)	3.4 (0-7)	4.2 (0.3-8.1)	0	73.1 (64.4-81.8)	0.5 (0-1.9)	0
**Spain**	427	146 (34.4%)	12.3 (5.9-18.7)	10.3 (4.3-16.3)	12.3 (5.9-18.7)	0.7 (0-2.3)	91.8* (86.4-97.2)	0	3.4 (0 -7.0)
**Sweden**	345	104 (30.1%)	2.9 (0-6.2)	2.9 (0-6.2)	2.9 (0-6.2)	0	73.1* (64.4-81.8)	2.0 (0-4.7)	0
**UK**			No children in study sample due to ethical considerations						

**Significant difference with adults under p < 0.05.

*Significant difference with adults under p < 0.1.

### Recommendations in treatment guidelines

Some guidelines were not complete in their coverage of all four infections for both adults and children. Since folliculitis and furuncle are related infections, they were often discussed together in guidelines and the same recommendations were applied. Overall, the first-choice recommendations for skin infections were very consistent across Europe. Almost all recommended first-choice antibiotics were of the B-lactam class, mainly flucloxacillin. Austria and Sweden also recommended cephalosporins for impetigo, folliculitis and furuncle; while in the Netherlands macrolides were preferred for cellulitis in children. For the treatment of impetigo, all guidelines recommended to first start treatment with a topical antibiotic (most often fusidic acid). The second-choice recommendations consisted of a wider range of antibiotics. Most countries used the same antibiotic for adults and children, with an adjusted dosage for children.

### Congruency of first- and second-choice antibiotics with AMR patterns

One can assess the congruency of the recommendations with AMR patterns by determining whether the resistance to the antibiotics is <20% (Tables 3 and 4) [28]. As previously mentioned low resistance to the topical agents was found in *S. aureus*, so the topical treatment recommendations were all congruent with AMR in the community.

**Table 3:** **Congruency of treatment recommendations for skin infections in adults with national commensal** ***S. aureus*** **resistance rates**

	**Topical AB**	Resistance rate*	**First choice systemic AB**	Resistance rate*	**Second choice systemic AB**	Resistance rate*
Impetigo						
**Austria**	Fusidic acid	1.0	Cephalosporin	No data	Amoxicillin + Clavulanic acid	1.5
**Belgium**	Fusidic acid	3.4	Flucloxacillin	2.2	Clarithromycin	16.3
**France**	Fusidic acid	4.0	No specific advice			
**Hungary**	No guideline					
**Netherlands**	Fusidic acid	5.2	Flucloxacillin	1.0	Azithromycin	6.9
**Spain**	Mupirocin	1.6	Penicillin (IM)/Cloxacillin	**86.0**	Clindamycin	9.5
				1.3		
**Sweden**	Retapamulin	0	Flucloxacillin	0	Cefadroxil	No data
**UK**	Fusidic acid	7.8	Flucloxacillin	1.6	Clarithromycin	8.9
Cellulitis						
**Austria**			Penicillin (parenteral)	**64.4**	Clindamycin	11.1
**Belgium**			Flucloxacillin	2.2	Clindamycin	14.4
**France**	No guideline					
**Hungary**	No guideline					
**Netherlands**			Flucloxacillin	1.0	Claritromycin	5.5
**Spain**			Cloxacillin	1.3	Amoxicillin + Clavulanic acid	1.3
**Sweden**	No guideline					
**UK**			Flucloxacillin	1.6	Ery/Clarithromycin	8.9
Folliculitis and Furuncle						
**Austria**			Cephalosporin	No data	Amoxicillin + Clavulanic acid	1.5
**Belgium**	No guideline					
**France**	No guideline					
**Hungary**	No guideline					
**Netherlands**			Flucloxacillin	1.0	No second choice	
**Spain**			Cloxacillin	1.3	No second choice	
**Sweden**			Flucloxacillin	0	Cefadroxil	No data
**UK**			Flucloxacillin	1.6	Ery/Clarithromycin	8.9

*A recommendation is congruent if the resistance rate in *S. aureus* to that antibiotic is <20%. Data in bold indicate a resistance rate of >20%.

**Table 4:** **Congruency of treatment recommendations for skin infections in children with national commensal** ***S. aureus*** **resistance rates**

	**Topical AB**	Resistance rate*	**First choice systemic AB**	Resistance rate*	**Second choice systemic AB**	Resistance rate*
Impetigo						
**Austria**	Fusidic acid	4.3	Cephalosporin	No data	Amoxicillin + clavulanic acid	0
**Belgium**	Fusidic acid	0	Flucloxacillin	0	Clarithromycin	16.6
**France**	No guideline					
**Hungary**	No guideline					
**Netherlands**	Fusidic acid	0.5	Flucloxacillin	0	Azithromycin	5.9
**Spain**	No guideline					
**Sweden**	Retapamulin	0	Cefadroxil	No data	Flucloxacillin	0
**UK**	Fusidic acid	No data	Flucloxacillin	No data	Clarithromycin	No data
Cellulitis						
**Austria**	No guideline					
**Belgium**			Flucloxacillin	0	No second choice	
**France**	No guideline					
**Hungary**	No guideline					
**Netherlands**			Clarithromycin	4.2	Azithromycin	5.9
**Spain**	No guideline					
**Sweden**	No guideline					
**UK**			Flucloxacillin	No data	Ery/Clarithromycin	No data
Folliculitis and Furuncle						
**Austria**			Cephalosporin	No data	Amoxicillin + Clavulanic acid	0
**Belgium**	No guideline					
**France**	No guideline					
**Hungary**	No guideline					
**Netherlands**	No guideline					
**Spain**	No guideline					
**Sweden**	No guideline					
**UK**			Flucloxacillin	No data	Ery/Clarithromycin	No data

*A recommendation is congruent if the resistance rate in *S. aureus* to that antibiotic is <20%.

#### Adults

All first choice recommendations for oral treatment were congruent with the AMR patterns. In Austria (for cellulitis and erysipelas) and Spain (for impetigo) a parenteral treatment with penicillin was advised, which was not congruent with the high penicillin resistance rates found in *S. aureus*. The second-choice antibiotic treatment recommendations were also congruent, with measured resistance levels of <20%, although for Belgium some recommended antibiotics exceeded 15% resistance. We found that all recommendations in the Swedish guidelines concerned antibiotics with an AMR level of 0%.

#### Children

Only oral therapy was advised for children, in most cases consisting of the same antibiotic that is used for adults (flucloxacillin) but with adjusted dosages. All recommended antibiotics showed a resistance level of <20% and were therefore congruent.

## Discussion

This study assessed the congruency of primary care treatment guidelines for bacterial skin infections with nasal AMR levels of *S. aureus* in the community in nine European countries.

### Congruency of recommendations

To assess the congruency of recommendations we used a threshold of 20%: antibiotics to which *S. aureus* has resistance rates of <20% are considered congruent [28]. Our study showed that most of the first- and second-choice recommendations in the treatment guidelines were congruent with AMR patterns in nasal *S. aureus* in the community, except for two recommendations for penicillin. Azithromycin was appropriate in the Netherlands, but the relatively high resistance rates in other countries (up to 30%) warrant a cautious use of this antibiotic for skin infections.

Given the resistance levels to penicillin in nasal *S. aureus* in the community, our findings suggest that it should not be used as a first- or second-choice antibiotic for *S. aureus* infections in primary care. Most guidelines for skin infections that we assessed were already congruent with this finding as they did not recommend the use of penicillin, except for two first-choice recommendations for penicillin in Austria and Spain. The penicillin recommendation for Austria was also used for erysipelas, which is often caused by a streptococcus. The same was true for non-bullous impetigo, for which penicillin was recommended in Spain [32]. Literature regarding AMR levels in streptococci indicates a high susceptibility for penicillin [33], implying these recommendations might be congruent as well in Austria and Spain.

### Strengths and limitations

The strength of our study is the broad scope of the data from nine countries across Europe (North, South, East and West), with a high variation in antibiotic use [5]. Our study is also unique as it assesses the congruency of treatment guidelines for *S. aureus* skin infections in primary care based on the prevalence of antibiotic resistance patterns. The treatment guidelines are issued nationally and have been supplied by experts who are aware of the most frequently used guidelines in their countries. Our study is complete by covering both recommendations for adults and for children.

A previous paper presented information on the dosage and duration of the treatment recommendations. Although the relationship between certain dosage-duration regimes and the development of resistance is not fully clear, it is noteworthy that the treatment guidelines from Sweden recommend higher dosages, while at the same time low AMR was observed [34].

One limitation of our study is that although it tested resistance to a wide range of antibiotics, not all antibiotic recommendations in the guidelines were covered on a one-on-one basis and in some cases we had to use the prevalence of resistance to a similar antibiotic [30,31]. Also, since we excluded patients with current infections or risk factors for AMR (antibiotic use or hospitalisation in the past 3 months), the level of AMR might be an underestimation for the total population in the community. However, given that antibiotic resistance can linger for up to one year [6], we assume our sample to be a good approximation of primary care patients.

Another limitation is the assumption we made that the AMR patterns found in nasal *S. aureus* are similar to those of pathogenic *S. aureus* found in SSTIs. To our knowledge, this relationship has not been conclusively studied, and future research might be able to fill this knowledge gap.

Our study is also limited in its choice of pathogen: we focussed on *S. aureus* due to its relatively high prevalence and impact on public health but skin infections can also be caused by a *Streptococcus* bacteria which may have other AMR patterns and would be relevant to also consider in treatment guidelines. However, since the main pathogen for these skin infections is *S. aureus* we emphasize its importance and recommend that resistance patterns of this pathogen are taken into account when updating or developing treatment guidelines for skin infections. Finally, although our study uses aggregated data, possible regional differences in AMR patterns of pathogens could also be integrated into empiric treatment guidelines.

### Implications for primary care

Most AMR studies present data from non-community settings (e.g. the hospital setting) [34] and there is limited data on antibiotic resistance in the community. The prevalence of resistance of *S. aureus* we found in primary care is lower than the levels reported in hospitals [4,35] and we recommend that recent national AMR data from the community should be taken into account to create more effective and evidence-based treatment guidelines for primary care. In such initiatives other factors affecting evidence-based practice, such as the implementation process or adherence to guidelines, should also be incorporated [36]. Evidence-based guidelines are, however, a first step to control the development of antibiotic resistance.

## Conclusions

Our comparison of primary care treatment guidelines with AMR patterns of commensal *S. aureus* in the community showed that not all European countries have developed national guidelines for the treatment of common skin infections in primary care and emphasizes the need to develop treatment guidelines in these countries. The first- and second-choice recommendations in the available treatment guidelines proved to be congruent with the national AMR patterns found in nasal colonized *S. aureus*: almost all recommendations concerned antibiotics to which *S. aureus* had low resistance levels (<20%). Given the high resistance to penicillin that has been demonstrated for commensal *S. aureus*, we recommend that this antibiotic should not be used in primary care treatment of *S. aureus* related bacterial skin infections. Based on the variation in antimicrobial resistance levels between countries, age groups and health care settings, national data regarding antimicrobial resistance in the community should be taken into account when updating or developing primary care treatment guidelines.

## Additional files

Additional file 1: Table S1. National primary care treatment guidelines included in this study. Additional file 2: Table S2. Comparison of antibiotics. Some antibiotics recommended in the treatment guidelines have not been tested for resistance in the APRES study. For these, we used the resistance rates of closely related antibiotics.
