# Supplementary material for: Primary care treatment guidelines for skin infections in Europe: congruence with antimicrobial resistance found in commensal Staphylococcus aureus in the community
Source: BMC Fam Pract. 2014 Oct 25;15:175. doi: 10.1186/s12875-014-0175-8 (PMC4220054; doi:10.1186/s12875-014-0175-8)
Supplement: Additional file 1: Table S1. — National primary care treatment guidelines included in this study. [file 12875_2014_175_MOESM1_ESM.doc]

**Additional file 1: Table S1 National primary care treatment guidelines included in this study**

| **Issuing country** | **Issuing**  **organisation** | **Title of guideline in English (link)** | **Year of publication** | **Number of guidelines** |
| --- | --- | --- | --- | --- |
| **Austria** | Verlagshaus der Ärzte | Evidence based medicine for use in clinical  and practice environment  (http://www.ebm-uidelines.at/index.php) | 2009 - 2012 | 3 |
| **Belgium** | Belgian Antibiotic Policy Coordination Committee (BAPCOC) | Belgian guide for anti-infectious treatment in  primary care  (http://www.bcfi.be/userfiles/File/antibioticagi  ds-NL.pdf) | 2012 | 2 |
| **Croatia** | No national guidelines for skin infections available | |  | **0** |
| **France** | French Society for General Medicine (SFMG) | Prescription of topical antibiotics in primary  and secondary skin infections  (http://www.infectiologie.com/site/medias/_  documents/consensus/2005-atb-locale  dermato-argu-afssaps.pdf) | 2004 | 1 |
| **Hungary** | Ministry of Health | The Ministry of Health protocol for Erysipelas  http://www.eum.hu/egeszsegpolitika/minoseg  fejlesztes/borgyogyaszat) | 2008 | 1 |
| **Netherlands** | Dutch College of General Practitioners (NHG) | Standards of the Dutch college of General  Practitioners – Bacterial Skin Infections M68  (http://www.nhg.artsennet.nl/standaarden/)  **Currently under revision* | 2007 | 1 |
| **Spain** | Medical College / Ministry of Health and Consumption | Guide to good clinical practice for antimicrobial  treatment in the community  (http://www.comsegovia.com/pdf/guias/GBPC  %20TTO%20ANTIMICROBIANO.pdf) | 2006 | 1 |
| **Sweden** | Swedish Medical Products Agency and Strama  (Swedish strategic program  against antibiotic resistance) | Pharmacological treatment of skin and soft  tissue infections  (http://www.strama.se/dyn//.244.60.77.html) | 2008 | 1 |
| **United Kingdom** | National Health Service (NHS) | Clinical Knowledge Summaries  (http://cks.nice.org.uk/) | 2011 - 2013 | 3 |

For the purpose of this study. the guidelines were accessed in April 2014
