# Supplementary material for: Primary care treatment guidelines for skin infections in Europe: congruence with antimicrobial resistance found in commensal Staphylococcus aureus in the community
Source: BMC Fam Pract. 2014 Oct 25;15:175. doi: 10.1186/s12875-014-0175-8 (PMC4220054; doi:10.1186/s12875-014-0175-8)
Supplement: Additional file 2: Table S2. — Comparison of antibiotics. Some antibiotics recommended in the treatment guidelines have not been tested for resistance in the APRES study. For these, we used the resistance rates of closely related antibiotics. [file 12875_2014_175_MOESM2_ESM.doc]

**Additional file 2: Table S2 Comparison of antibiotics. Some antibiotics recommended in the treatment guidelines have not been tested for resistance in the APRES study. For these,, we used the resistance rates of closely related antibiotics.**

| **Antibiotic in guidelines** | **ATC code** | **Compared with antibiotic** | **ATC code** |
| --- | --- | --- | --- |
| Cephalosporins | J01D | No comparison because of wide range | |
| Amoxicillin | J01AC04 | Penicillin | J01CE0X |
| Amoxicillin+ Clavulanic acid | J01CR02 | Oxacillin | J01CF04 |
| Cloxacillin | J01CF02 | Oxacillin | J01CF04 |
| Flucloxacillin = Isoxazolylpenicillin | J01CF05 | Oxacillin | J01CF04 |
| Dicloxacillin | J01CF01 | Oxacillin | J01CF04 |
| Retapamulin | D06AX13 | Mupirocin | D06AX09 |
| Claritromycin | J01FA09 | Erythromycin | J01FA01 |
